# A model to simulate cardio-respiratory responses to fentanyl analgesia after traumatic injury

**DOI:** 10.3389/fphys.2026.1907767

**Published:** 2026-09-04

**Authors:** Varghese Kurian, Xin Jin, Anders Wallqvist, Jaques Reifman, Sridevi Nagaraja

**Affiliations:** 1 Department of Defense Biotechnology High Performance Computing Software Applications Institute, Defense Health Agency Research & Development, Medical Research and Development Command, Fort Detrick, MD, United States; 2 The Henry M. Jackson Foundation for the Advancement of Military Medicine, Inc., Bethesda, MD, United States

**Keywords:** cardio-respiratory model, combat care, fentanyl analgesia, hemorrhage, pain management, pharmacokinetic-pharmacodynamic

## Abstract

Prehospital pain management is challenging and is expected to become even more complex in future large-scale combat operations, where mass-casualty events and evacuation delay may be inevitable. An improved understanding of how analgesic drugs affect the physiological response of trauma patients can enhance treatment efficacy of combat casualties. We previously developed and validated a cardio-respiratory (CR) model for humans that accounts for vital-sign responses to hemorrhagic injuries, resuscitation with six fluid types, airway obstruction, and ketamine analgesia. Here, we extended the model to include the effect of fentanyl on vital signs by integrating existing fentanyl pharmacokinetic-pharmacodynamic models with the neuronal controller of the model. We calibrated and validated the extended model using experimental data from eight studies involving intravenous fentanyl administration (0.71–50.00 μg/kg) to healthy humans and swine with hemorrhagic injury. The model predictions reasonably captured the trend of the experimental data, with root mean square errors (RMSEs) between model predictions and measured data of 0.83 L/min for minute ventilation (MV), 1.20 mmHg for end-tidal carbon dioxide, 0.09 L for tidal volume, and 1.61 mmHg for mean arterial pressure, all of which were within 3–12% of their baseline values. For plasma fentanyl concentration, we obtained RMSEs of 0.79 μg/L in humans and 25.83 μg/L in swine. In simulations, we observed that as hemorrhage increased from 0 to 40% of blood volume, the fentanyl-induced decrease in MV increased from 19 to 34% of its baseline value prior to administration due to reduced fentanyl clearance. Similarly, in simulations of airway obstruction, the fentanyl-induced decrease in MV was 26% of its baseline value after a 100% obstruction compared to only 19% for a no-obstruction condition. Given that most combat casualties receive either fentanyl or ketamine for pain management, the ability to predict and quantify the physiological effects of these drugs will allow us to generate relevant synthetic datasets of diverse battlefield scenarios.

## 1 Introduction

Pain management is a crucial aspect of battlefield trauma, with ~70% of U.S. combat casualties receiving analgesics at the point of injury (POI) or during evacuation (Schauer et al., 2017). While effective, analgesics like ketamine and fentanyl are known to alter cardio-respiratory variables, including mean arterial pressure (MAP) and minute ventilation (MV), in healthy individuals (Mildh et al., 1998; Xiang et al., 2020). Although these effects may be further complicated by the physiological stress of trauma, they have not been systematically quantified. To care for casualties on the battlefield, combat medics must be able to adequately account for drug-induced vital-sign changes to avoid misinterpreting a casualty’s true physiological status. Artificial intelligence (AI)-powered decision-support systems can help mitigate this challenge by assisting medics in monitoring, prioritizing, and treating casualties at the POI (Jin et al., 2024; Subramaniyan et al., 2025; Burke et al., 2026). However, a major roadblock in deploying these systems is the lack of large amounts of high-quality labeled data that capture the combined physiological impact of traumatic injuries and subsequent interventions, including analgesia (Quinn et al., 2024; Holcomb et al., 2025). Because real-world clinical or animal trauma data are difficult to acquire, simulations using validated mathematical models offer an effective alternative for generating synthetic data that replicate human physiological responses to battlefield injuries.

Because physiological responses to trauma and treatment modalities vary considerably between individuals, personalizing mathematical models to build cardiovascular digital twins could further enhance combat medics’ ability to care for casualties. Using established approaches like Kalman filtering, lower-order mechanistic models comprised of a manageable number of model parameters can be calibrated to track individual vital-sign trajectories and predict responses to interventions. We previously used this strategy to optimize alertness impairment through personalized caffeine and sleep recommendations (Vital-Lopez et al., 2023). When interfaced with wearable sensors, such digital twin systems could ingest continuous streams of vital-sign data to provide real-time tracking and precise, individualized treatment recommendations.

While certain validated fentanyl pharmacokinetic (PK)-pharmacodynamic (PD) models incorporate the empirical dependence of fentanyl kinetics on cardiac output during hemorrhagic trauma or burn injuries (Mogil, 2009; Carbone, 2019; Lie and Rognes, 2025), PK-PD models of fentanyl-induced vital-sign responses are currently available only for healthy individuals (Egan et al., 1999; Mildh et al., 2001; Han et al., 2007; Bienert et al., 2020). Furthermore, several mechanistic cardio-respiratory models that predict the effect of hemorrhagic injuries and fluid resuscitation on vital signs do not incorporate fentanyl’s effect on them (Mardel et al., 1995; Drobin and Hahn, 1999; Tatara et al., 2007; Curcio et al., 2021; Jin et al., 2023; Kurian et al., 2025a). Currently, no quantitatively validated models exist to predict fentanyl-induced cardio-respiratory changes following the most common traumatic injuries, such as hemorrhage and airway obstruction, which represent ~99% of survivable battlefield deaths (Eastridge et al., 2012).

Recently, our U.S. Department of Defense team developed and validated the cardiorespiratory (CR) model to capture human vital-sign responses to hemorrhage, airway obstruction, respiratory perturbations, resuscitation with six fluid types, and ketamine analgesia (Jin et al., 2023, 2025; Kurian et al., 2025a, 2025b). Here, we extended and validated the mechanistic CR model by integrating it with an existing fentanyl PK-PD model to incorporate the effects of fentanyl analgesia on multiple vital signs, including MAP, MV, end-tidal carbon dioxide (ETCO_2_), respiratory rate (RR), tidal volume (V_T_), cardiac output (CO), and heart rate (HR). Given that different studies have successfully used this approach to investigate fentanyl-induced vital-sign changes during non-analgesic dosing, specifically during anesthesia recovery and overdose treatment (Magosso et al., 2004; Clipp et al., 2016; Mann et al., 2022; Baird et al., 2024), here we hypothesized that the incorporation of PK-PD models will also work for analgesic fentanyl doses. Specifically, we first integrated the MV output from an existing fentanyl PK-PD model within the CR model by modifying one of its feedback controllers. We then calibrated and validated the model using seven human studies and one animal study involving 0.71 to 50.00 μg/kg fentanyl boluses. Finally, we used the validated CR model to investigate fentanyl analgesia following moderate to severe hemorrhage and 100% airway obstruction *in silico*. These analyses allowed us to generate experimentally testable hypotheses regarding the effects of fentanyl analgesia on vital signs after traumatic injuries.

## 2 Materials and methods

### 2.1 Computational model

**Figure 1:**
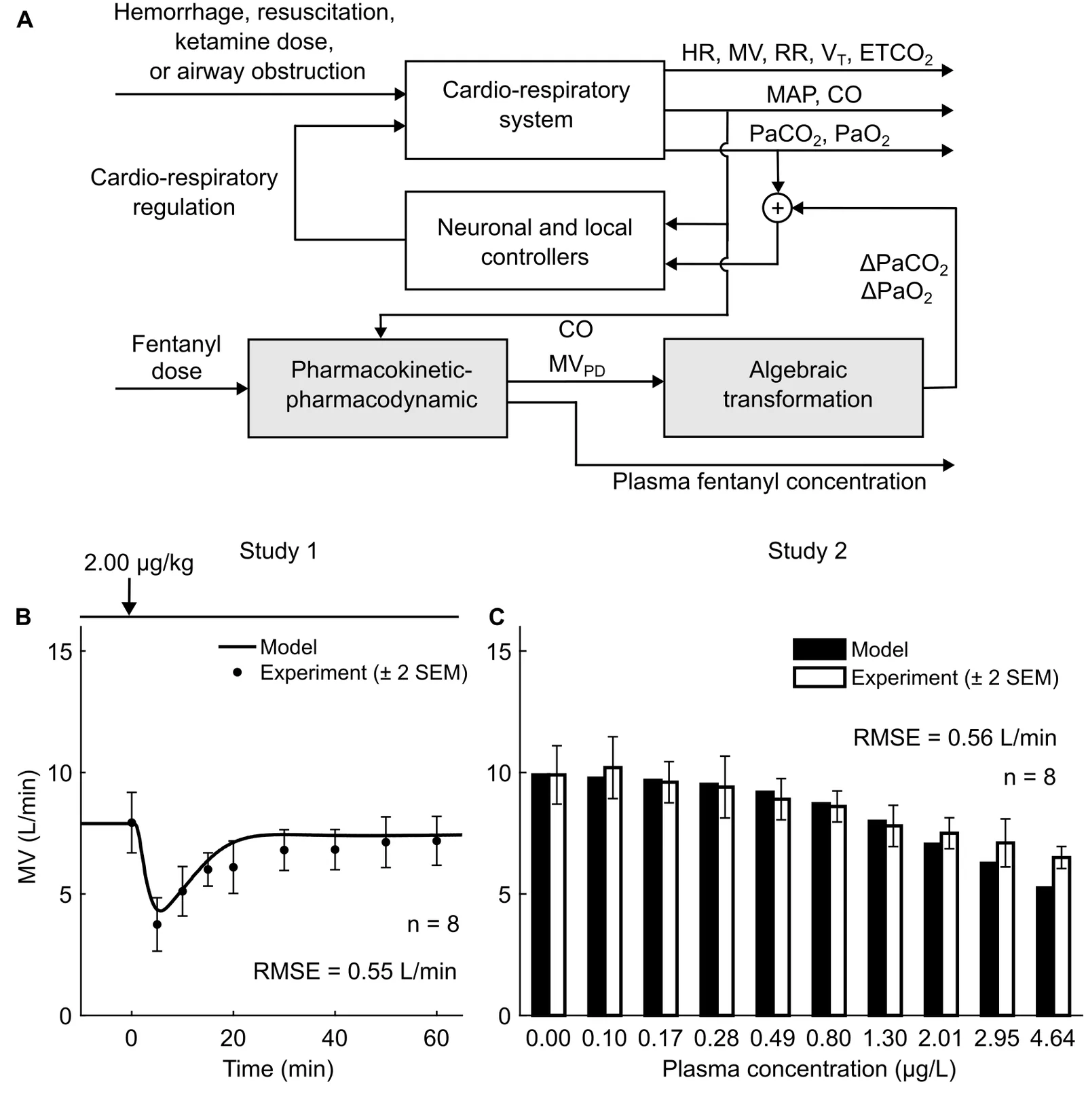
**(A)** Schematic showing the integration of the fentanyl pharmacokinetic-pharmacodynamic model into the cardio-respiratory (CR) model, with the shaded boxes indicating the newly added extensions. **(B)** Comparison of the experimental time-dependent minute ventilation (MV) data from *Study 1* with the corresponding model calibration. Error bars denote two standard errors of the mean (SEM). The timeline at the top illustrates the time and dose of bolus fentanyl administration. **(C)** Comparison of the experimental steady-state MV data from *Study 2* with the corresponding model calibration across different plasma fentanyl concentrations. CO, cardiac output; ETCO_2_, end-tidal carbon dioxide; HR, heart rate; MAP, mean arterial pressure; PaCO_2_, partial pressure of arterial carbon dioxide; PaO_2_, partial pressure of arterial oxygen; RMSE, root mean square error; RR, respiratory rate; V_T_, tidal volume.

The previously developed CR model represents the human cardiovascular and respiratory systems and their regulatory mechanisms, to predict changes in the time course of vital signs in response to hemorrhagic injuries, airway obstruction, fluid resuscitation, and ketamine administration (Jin et al., 2023, 2025; Kurian et al., 2025a, b). Here, we used a two-step process (**Figure 1A**, shaded boxes) to extend this model and incorporate the effects of fentanyl on the model-predicted vital signs. In the first step, we used a pharmacokinetic PK-PD model that took fentanyl dose and the CR-model-predicted CO as inputs and provided as outputs the time course of plasma fentanyl concentration and the resulting MV, which we considered as the target value (MV_PD_) that represented a fentanyl-induced reduction in MV. In the second step, we used an algebraic equation to compute changes in two inputs of a neuronal controller, i.e., arterial partial pressure of oxygen (ΔPaO_2_) and carbon dioxide (ΔPaCO_2_), required to drive the CR model prediction of MV to match the target value computed by the PK-PD model so that the predicted MV equaled MV_PD_.

#### 2.1.1 Fentanyl PK-PD model

To compute changes in the plasma fentanyl concentration, we used the two-compartment PK model originally developed by Yassen et al. (2007) for healthy human described in Equations 1 and 2 below:

(1) $${\text{V}}_{\text{c}}\frac{\text{d}\left[{\text{C}}_{\text{c}}\right]}{\text{dt}}=-\text{Cl}\cdot \left[{\text{C}}_{\text{c}}\right]-\text{Q}\left(\left[{\text{C}}_{\text{c}}\right]-\left[{\text{C}}_{\text{p}}\right]\right)+{\text{R}}_{\text{in}}$$

(2) $${\text{V}}_{\text{p}}\frac{\text{d}\left[{\text{C}}_{\text{p}}\right]}{\text{dt}}=\text{Q}\left(\left[{\text{C}}_{\text{c}}\right]-\left[{\text{C}}_{\text{p}}\right]\right)$$

where V_c_ and V_p_ represent the volume of the central compartment (blood and highly perfused tissues) and peripheral compartment (less perfused tissues), respectively, [C_c_] and [C_p_] represent the fentanyl concentration in the central and peripheral compartments, respectively, Cl and Q denote the elimination and intercompartmental clearances of fentanyl, respectively, and R_in_ represents the intravenous fentanyl administration rate. This model assumed instantaneous distribution of fentanyl within each compartment and that no elimination occurred from the peripheral compartment.

Moreover, to account for the shift in fentanyl pharmacokinetics under pathological conditions (Bienert et al., 2020), such as hemorrhagic injury, we represented Cl, V_c_, Q, and V_p_ in Equations 1 and 2 as empirical functions of CO, as follows:

(3) $$\text{Cl}={\text{Cl}}_{0}\left(1+\theta_{\text{Cl}}\frac{\text{CO}-{\text{CO}}_{\text{B}}}{{\text{CO}}_{\text{B}}}\right)$$

(4) $${\text{V}}_{\text{c}}={\text{V}}_{\text{c}0}\left(1+\theta_{\text{Vc}}\frac{\text{CO}-{\text{CO}}_{\text{B}}}{{\text{CO}}_{\text{B}}}\right)$$

(5) $$\text{Q}={\text{Q}}_{0}\left(1+\theta_{\text{Q}}\frac{\text{CO}-{\text{CO}}_{\text{B}}}{{\text{CO}}_{\text{B}}}\right)$$

(6) $${\text{V}}_{\text{p}}={\text{V}}_{\text{p}0}\left(1+\theta_{\text{Vp}}\frac{\text{CO}-{\text{CO}}_{\text{B}}}{{\text{CO}}_{\text{B}}}\right)$$

where the subscript “0” represents the PK model parameters in healthy individuals (i.e., when CO = CO_B_), CO_B_ represents the baseline value of CO (5 L/min), and θ denotes the gain used to quantify the CO-induced shift in the respective kinetic parameter. Leveraging the work of Han et al. (2007), we adapted Equations 3-6 from a swine study by Birkholz et al. (2018) and adjusted the gains θ to reflect the corresponding fentanyl-induced kinetics shift in humans. Briefly, Han et al. (2007) estimated the four PK model parameters, Cl, V_c_, Q, and V_p_, in Equations 1 and 2 using two distinct human datasets, where subjects received a bolus dose (2.86 μg/kg) of fentanyl: one based on healthy (non-cardio compromised) controls and the other based on burn patients whose CO was, on average, ~62% higher than controls. Accordingly, we estimated the gains for humans by solving Equations 3-6 for θ_Cl_, θ_Vc_, θ_Q_, and θ_Vp_, where we substituted the pathological/healthy ratio of the four PK model parameters (e.g., Cl/Cl_0_ in Equation 3) by the associated burn/healthy ratio estimated by Han et al. (2007) and assumed CO_B_ and CO to take their corresponding baseline and 1.62 times baseline values, respectively.

We used the PD model developed by Olofsen et al. (2022) to compute the fentanyl-induced decrease in MV (i.e., MV_PD_) using Equations 7 and 8 below:

(7) $$\frac{\text{d}\left[{\text{C}}_{\text{e}}\right]}{\text{dt}}={\text{k}}_{\text{eo}}\left(\left[{\text{C}}_{\text{c}}\right]-\left[{\text{C}}_{\text{e}}\right]\right)$$

(8) $${\text{MV}}_{\text{PD}}={\text{MV}}_{\text{B}}\left(1-\frac{\left[{\text{C}}_{\text{e}}\right]}{{\text{C}}_{50}+\left[{\text{C}}_{\text{e}}\right]}\right)$$

where [C_e_] represents the concentration of fentanyl at its effect site, k_eo_ denotes the equilibration rate constant, MV_B_ denotes the baseline MV (6.62 L/min), and C_50_ represents the fentanyl concentration resulting in half-maximal respiratory depression. Although the Olofsen et al. model was developed under iso-hypercapnic and iso-normic conditions, we selected it because its fentanyl dose range (1.07–3.57 μg/kg) aligns closely with the experimental scenarios investigated herein. While more complex mechanistic PK-PD models exist that predict PaO_2_ and PaCO_2_ in addition to MV for similar or higher fentanyl doses (Magosso et al., 2004; Mann et al., 2022; van Lemmen et al., 2025), we achieved comparable mechanistic feedback by embedding the PK-PD output within the CR model’s neuronal controllers, as described in Subsection 2.1.2 below.

Finally, the original PK-PD models were developed for healthy humans. The modifications we made to the PK model herein allowed us to consider fentanyl concentrations under pathological conditions in humans. However, we assumed that the fentanyl-driven changes in MV (in the PD model and Equations 7, 8) that were derived using data from healthy individuals also hold for individuals who suffered hemorrhagic and airway obstruction trauma.

#### 2.1.2 Incorporation of PK-PD model outputs into the CR model

Because MV is an output of both the CR model and the PK-PD model, we integrated these components by using the PK-PD predicted MV (MV_PD_) as a target for the CR model’s neuronal controller. To achieve this, we implemented an algebraic transformation (**Figure 1A**) to compute the specific incremental changes in PaO_2_ and PaCO_2_ required to drive the CR model’s MV to match MV_PD_ (Magosso et al., 2004; Kurian et al., 2025b; van Lemmen et al., 2025). Specifically, at each time step, we added the changes ΔPaO_2_ and ΔPaCO_2_ to the current CR-model-predicted endogenous PaO_2_ and PaCO_2_, respectively, before providing them as inputs to the neuronal controller. In response, the controller adjusted the RR and respiratory muscle pressure to drive the model-predicted MV to match MV_PD_. To develop the transformation, we performed simulations with the CR model using constant ΔPaO_2_ and ΔPaCO_2_ values as inputs and recorded the resulting steady-state value of MV. We performed these simulations for 43 pairs of ΔPaO_2_ and ΔPaCO_2_ from 0 to 84% of their respective baseline values (PaO_2,B_ = 90 mmHg and PaCO_2,B_ = 40 mmHg), at 2% increments. While fentanyl affects both PaO_2_ and PaCO_2_ (Pattullo, 2022), its specific contribution to each of them has not been quantified, therefore we assumed that fentanyl affected PaO_2_ and PaCO_2_ equally relative to their baseline values. Using these simulated data, we fitted a quadratic equation between the relative PaO_2_ (ΔPaO_2_/PaO_2,B_) and PaCO_2_ (ΔPaCO_2_/PaCO_2,B_) changes, which we denoted as Pgas_ss_, and the target MV_PD_, as described in Equation 9 below:

(9) $${\text{Pgas}}_{\text{ss}}={\text{c}}_{0}+{\text{c}}_{1}\left(\frac{{\text{MV}}_{\text{PD}}}{{\text{MV}}_{\text{B}}}\right)+{\text{c}}_{2}{\left(\frac{{\text{MV}}_{\text{PD}}}{{\text{MV}}_{\text{B}}}\right)}^{2}$$

where c_0_, c_1_, and c_2_ represent the coefficients of the quadratic function. To confirm the goodness-of-fit of the quadratic model in Equation 9, we computed the coefficient of determination *R^2^* and obtained a value of 0.99. Finally, once we determined Pgas_ss_ and the corresponding steady-state values for ΔPaO_2_ and ΔPaCO_2_ needed to achieve a specific MV_PD_, we did not directly provide them as inputs to the controller because abrupt input changes cause oscillations in its response. Instead, we used Equations 10 and 11 to generate a gradual change in these values by applying a smoothing parameter k_s,gas_, as shown below:

(10) $$\frac{\text{d}\Delta {\text{PaO}}_{2}}{\text{dt}}={\text{k}}_{\text{s,gas}}\left({\text{PaCO}}_{2, B}\cdot {\text{Pgas}}_{\text{ss}}-\text{Δ}{\text{PaO}}_{2}\right)$$

(11) $$\frac{\text{d}\Delta {\text{PaCO}}_{2}}{\text{dt}}={\text{k}}_{\text{s,gas}}\left(\text{Δ}{\text{PaCO}}_{2}-{\text{PaCO}}_{2, B}\cdot {\text{Pgas}}_{\text{ss}}\right)$$

The extended CR model consisted of 148 ordinary differential and algebraic equations and 177 associated parameters. The model inputs included the rate of hemorrhage, the infusion rate and type of resuscitation fluid, the rates of fentanyl and ketamine administration, the concentrations of O_2_ and CO_2_ in inspired air, the airway opening pressure, and the fraction and location of airway obstruction. Depending on the simulation, some input values were set to zero. For example, if we did not simulate hemorrhage or fluid resuscitation, we set those inputs to zero. Simulation times varied according to the specific protocols of the experimental studies we simulated, ranging from 45 minutes (*Study 7*) to 600 minutes (*Study 8*). The model outputs included the mean, systolic (SBP), and diastolic (DBP) arterial blood pressures, HR, CO, MV, V_T_, RR, ETCO_2_, and blood gas concentrations. We solved the model equations using Euler’s method (Griffiths and Higham, 2010) with a time step of 4.17×10^−4^ min and performed all simulations using MATLAB 24.2 (MathWorks, Natick, MA).

### 2.2 Model calibration

We calibrated three model parameters. First, we calibrated the two PD parameters k_eo_ and C_50_ in Equations 7 and 8, and after integration of the calibrated PK-PD model with the CR model, we calibrated the smoothing parameter k_s,gas_ in Equations 10 and 11. Because the derivation of k_eo_ and C_50_ was based on experiments where volunteers inhaled air different from atmospheric air (Olofsen et al., 2022), we calibrated them using data reported in *Studies 1* and *2* (**Table 1**) where healthy volunteers inhaled atmospheric air and received either a bolus fentanyl dose of 2.00 μg/kg or a continuous fentanyl administration that maintained a steady-state plasma fentanyl concentration between 0.10 and 4.64 μg/L (Mildh et al., 1998, 2001). To identify the optimal parameter values from within their respective physiological ranges (Mildh et al., 2001; Yassen et al., 2007; Algera et al., 2021; Olofsen et al., 2022), we performed a global optimization to minimize the root mean square error (RMSE) between the PK-PD model-predicted MV and the experimental data. Specifically, we generated eight sets of initial guesses for the two parameters, and using the MATLAB *fminsearchbnd* function, we optimized the values of the two parameters to minimize the RMSE between the predicted MV and the experimental data. We selected the parameter set that yielded the absolute lowest RMSE across all eight iterations as the final value. Because baseline MV values varied across studies, we normalized the MV predictions before comparing them with the experimental data by multiplying the predicted MV by the ratio of their experimental to predicted values at baseline (Jin et al., 2023). Finally, we followed the same approach for the k_s,gas_ parameter, testing two different initial guesses and minimizing the RMSE between the extended CR model-predicted MV (after normalization) and the corresponding data from *Study 1*.

**Table 1:** Studies used for calibration and validation of the extended cardio-respiratory (CR) model.

Study no.	No. subjects per group	Fentanyl dose (μg/kg)	Root mean square error (RMSE)						Reference
			Plasma fentanyl concentration (μg/L)	MV (L/min)	ETCO_2_ (mmHg)	RR (breaths/min)	V_T_ (L)	MAP (mmHg)	
RMSE validation threshold^*^			0.52	1.48	1.76	2.51	0.21	6.48	
Studies used for calibration of the pharmacodynamic model and the algebraic transformation component									
1	8	2.00	–	0.55	–	–	–	1.32	Mildh et al., 1998
2	8	0.00–4.64^a^	–	0.56	–	–	–	–	Mildh et al., 2001
Studies used for validation of the extended CR model									
1^b^	8	2.00	–	0.83	–	–	–	1.61	Mildh et al., 1998
3	s13	1.07–3.57	0.83	–	–	–	–	–	Olofsen et al., 2022
4	5	1.43	0.76	–	–	–	–	–	Mather et al., 1998
5	12	0.71–1.43	0.18	0.83	0.94	1.27	0.09	–	van Lemmen et al., 2025
6	10	1.00–4.00	1.45	–	1.45	–	–	–	Bailey et al., 1990
7	10	3.00	–	–	–	1.07	–	–	Palkama et al., 1998
Studies used for validation of fentanyl pharmacokinetics during hemorrhage									
8^c^	8	50.00	Control: 13.20	–	–	–	–	–	Egan et al., 1999
			Hemorrhage: 38.46					–	

ETCO_2_, end-tidal carbon dioxide; MAP, mean arterial pressure; MV, minute ventilation; RR, respiratory rate; V_T_, tidal volume.

^a^Plasma concentration in μg/L.

^b^A group of *Study 1* participants received a 0.25-mg/kg dose of ketamine combined with a 2.00-μg/kg dose of fentanyl. We did not use these data for model calibration.

^c^Swine subjects.

^*^We computed the validation thresholds for each predicted output for *Studies 1* and *3–7* by calculating the mean of the two standard errors of the mean across all the experimental studies that reported the specific output.

### 2.3 Model validation

We validated the extended CR model under healthy and hemorrhagic conditions by comparing the model predictions of vital signs and plasma fentanyl concentration with previously reported experimental data from *Studies 3–8* and a subset of *Study 1* (*Study 1^b^* in **Table 1**) that we did not use for model calibration (Bailey et al., 1990; Mather et al., 1998; Mildh et al., 1998; Palkama et al., 1998; Egan et al., 1999; Olofsen et al., 2022; van Lemmen et al., 2025). From *Study 1^b^*, we used the vital-sign responses to a 60-s administration of 2.00 μg/kg fentanyl mixed with 0.25 mg/kg ketamine in healthy volunteers. *Studies 3–6* reported time courses of plasma fentanyl concentration and *Studies 5–7* reported the different vital signs in healthy volunteers after 1–5 intravenous fentanyl boluses ranging from 0.71 to 4.00 μg/kg. From *Study 8*, we used the plasma fentanyl concentration measured in two swine groups, which received 50 μg/kg of fentanyl administered over a 5-min interval, where one group served as control and the other underwent hemorrhage with ~42% of blood volume (BV) loss.

To simulate these experimental scenarios, we provided the corresponding hemorrhage and drug administration rates as inputs to the CR model and predicted the time courses of plasma fentanyl concentration and vital signs. To validate the model, we first normalized the model predictions using the same procedure as described in model calibration above and then computed the RMSEs between the normalized model predictions and the corresponding experimental data. In the absence of standardized RMSE thresholds for the comparisons shown in our study, we defined an acceptance criterion based on the inherent variability of the experimental data (Potter et al., 2020; Singh et al., 2026). Specifically, we defined a threshold for each vital sign and fentanyl concentration by calculating the mean of the two standard errors of the mean (SEM) across the corresponding human validation studies. For the swine study involving hemorrhage, we defined separate thresholds for the control and hemorrhage groups by calculating the mean of the two SEM for each group. We considered a prediction “acceptable” if the RMSE remained below its respective threshold. In addition, we computed the percentage of normalized model predictions that fell within ±2 SEM of the experimental data.

### 2.4 *In silico* analysis of the physiological effects of analgesia under hypovolemia and airway obstruction

We used the validated CR model to investigate vital-sign responses to fentanyl and ketamine administration, individually or in combination, during hemorrhagic injury and airway obstruction. Because we could not identify previous large-animal or human studies that simultaneously considered traumatic injuries and fentanyl (or ketamine) administration, we arbitrarily defined and simulated two sets of scenarios.

In the first set of scenarios, we first simulated the experimental hemorrhage protocol described in Blackburn et al. (2021), where they challenged anesthetized swine with a 25% BV hemorrhage over 20 min with no analgesic administration and reported the time courses of ETCO_2_, MAP, and HR. We previously showed that the CR model could adequately predict these vital signs (Jin et al., 2025). Next, we repeated this simulation by adding intravenous administration of fentanyl or ketamine. At 2 min after the end of hemorrhage, we provided the first dose of an analgesic [either only fentanyl (0.75 μg/kg) or only ketamine (0.25 mg/kg) or a mixture of fentanyl (0.38 μg/kg) and ketamine (0.13 mg/kg) for a 70-kg human] and repeated this administration two more times, every 30 min. Finally, we repeated the entire simulation for a 40% BV hemorrhage, where we recorded the model-predicted time courses of MAP and MV for all scenarios.

In the second set of scenarios, we first simulated the experimental airway obstruction protocol described in Blackburn et al. (2019), where they subjected anesthetized swine to slow and rapid airway obstructions without analgesia, with both cases involving a 100% airway obstruction maintained for 3 min. They reported the time courses of MAP, SBP, DBP, HR, oxygen saturation (SpO_2_), and ETCO_2_, a subset of which we previously used to validate the CR model (Jin et al., 2025). We repeated this simulation by adding intravenous analgesic administration. Starting immediately after the end of each 100% airway obstruction, we applied the same three analgesia protocols as we did for hemorrhagic injuries and recorded the model-predicted time courses of SBP and MV for all scenarios.

In both sets of scenarios, we selected the fentanyl and ketamine doses, administration times (1 min), and time intervals between two consecutive doses (≥30 min) based on the Tactical Combat Casualty Care (TCCC) guidelines (Committee on Tactical Combat Casualty Care, 2024).

## 3 Results

### 3.1 Calibration of the PD model and the algebraic transformation

We calibrated two parameters (k_eo_ and C_50_) of the PD model and one parameter (k_s,gas_) of the algebraic transformation component using data from *Studies 1* and *2* (**Table 1**), which reported MV responses of humans to fentanyl analgesia. **Figures 1B, C** show the calibrated model’s MV predictions (solid line and solid bars, respectively) and the corresponding experimental data (solid circles and open bars, respectively). The calibrated model captured the general trend of the experimental data, i.e., within 5 min of a 2.00-μg/kg bolus fentanyl administration, MV decreased by 45% of its baseline value and gradually returned to within 10% of its baseline ~15 min after fentanyl administration and yielded an average RMSE of 0.55 L/min (**Figure 1B**). In addition, for scenarios involving continuous fentanyl administration to maintain steady-state plasma concentrations of 0.10–4.64 μg/kg, the calibrated model predicted steady-state MV with an RMSE of 0.56 L/min (**Figure 1C**). Finally, 94% of the calibrated model outputs fell within two SEM of the experimental data, indicating that the model results were generally indistinguishable from the mean values of the experimental measurements.

### 3.2 Validation of the extended CR model

**Figure 2:**
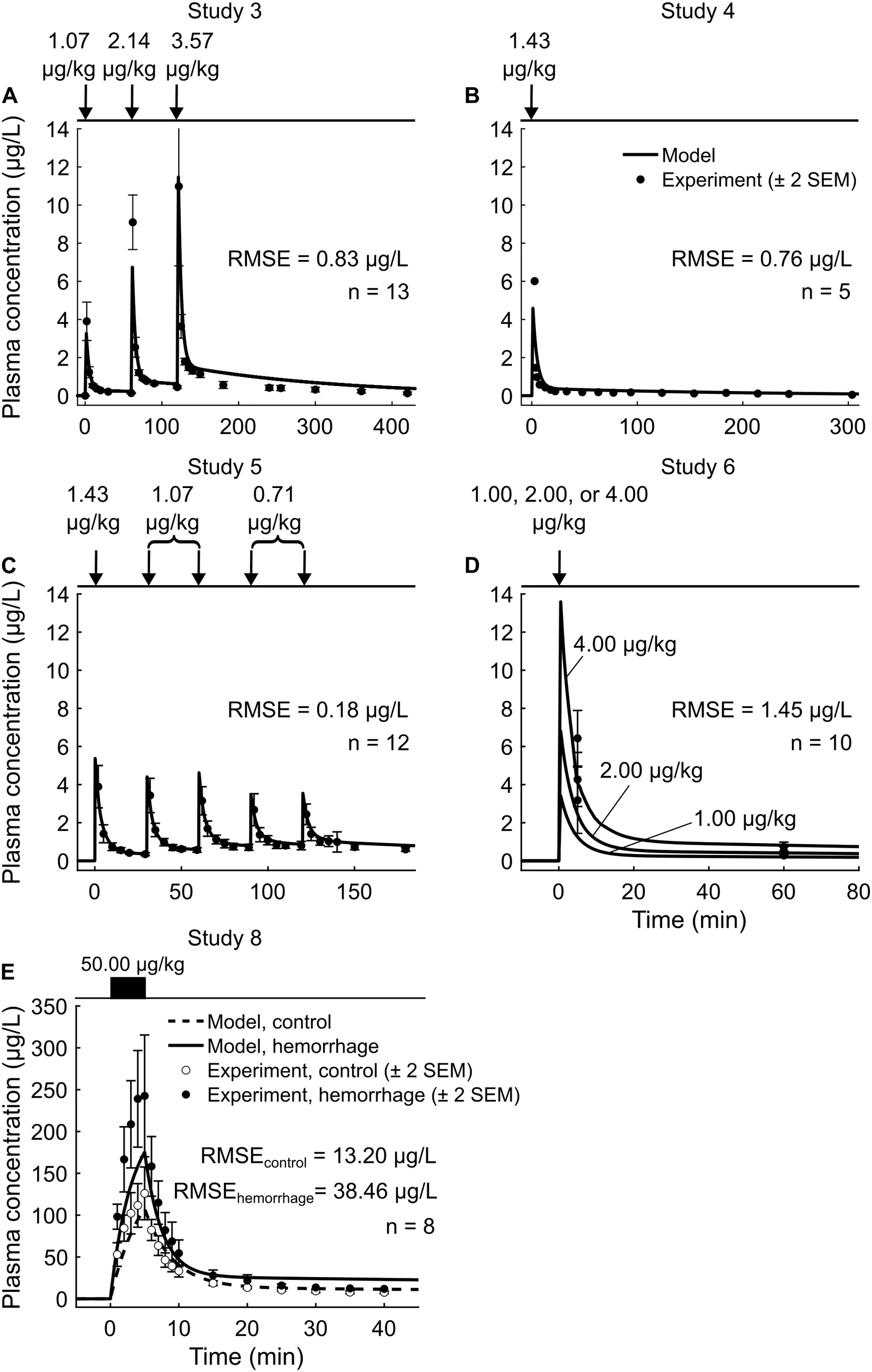
Comparison of the extended cardio-respiratory (CR) model predictions of plasma fentanyl concentration **(A*−*E)** with the corresponding experimental data from *Studies 3−6* and *8*. The arrows and the solid bar at the top of each panel illustrate the time and dose for each bolus or the slow, 5-min fentanyl administration of the experimental protocols. *Studies 3−6* involved healthy volunteers. *Study 8* involved two swine groups, a control group and a group subjected to ~42% blood volume hemorrhage. For *Study 5*, we show only the first 180 min for visualization purposes, however, we used the entire 242-min dataset for calculating the root mean square error (RMSE) and validation threshold. *Study 6* tested three different doses of fentanyl (1.00, 2.00, or 4.00 μg/kg), and we reported the overall RMSE across all three doses. Error bars denote two standard errors of the mean (SEM).

**Figure 3:**
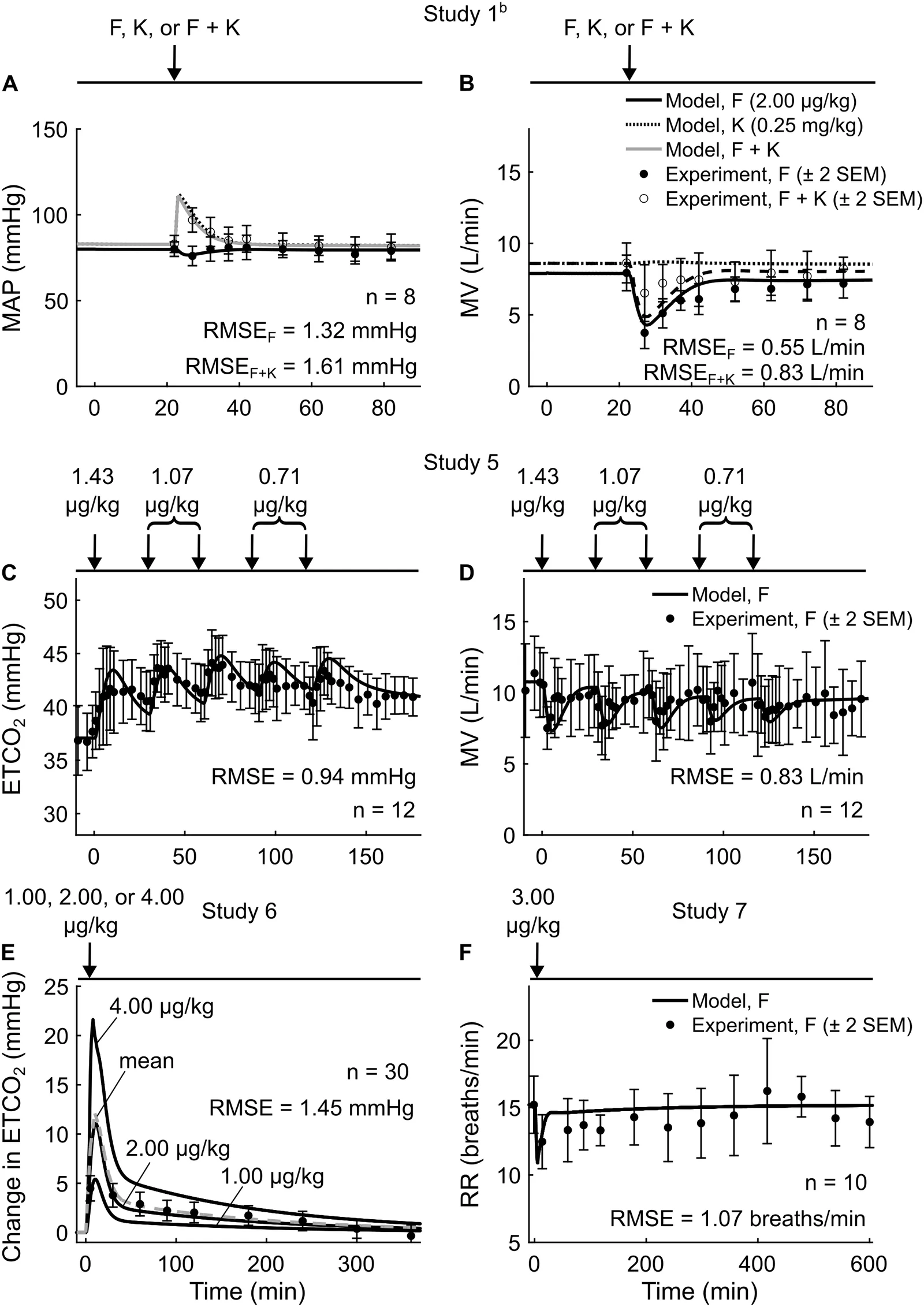
Comparison of the extended cardio-respiratory (CR) model vital-sign predictions with the corresponding experimental data from *Studies 1^b^, 5–7*. **(A)** Mean arterial pressure (MAP). **(B, D)** Minute ventilation (MV). **(C)** End-tidal carbon dioxide (ETCO_2_). **(E)** Change in ETCO_2_. **(F)** Respiratory rate (RR). The arrows at the top of each panel illustrate the time and dose of each bolus drug administration for the experimental protocols. For *Study 5*, we show only the first 180 min for visualization purposes, however, we used the entire 242-min dataset for calculating the root mean square error (RMSE) and validation threshold. *Study 6* tested three different doses of fentanyl (1.00, 2.00, or 4.00 μg/kg, model predictions shown by the solid black lines) but only reported the mean ETCO_2_ across the three groups (shown by the dashed gray line). We used the MV data of *Study 1* following fentanyl administration (solid circles) for model calibration and MAP and MV data following combined fentanyl and ketamine administration (open circles) for model validation. Error bars denote two standard errors of the mean (SEM). K, ketamine.

To validate the extended CR model, we compared its predictions of plasma fentanyl concentration, MAP, MV, ETCO_2_, RR, and V_T_ with the corresponding experimental data from *Studies 1^b^* and *3–8* (**Table 1**; **Figures 2**, **3**; **Supplementary Figure S1**; solid lines vs. solid circles, respectively). Overall, the model predictions captured the general trend of experimental data across the studies. For example, in experiments involving healthy individuals (*Studies 1^b^* and *3–7*), within 2 min of fentanyl administration of doses ranging from 0.71 to 4.00 μg/kg, the model-predicted plasma concentrations reached their peak and decreased to ~10% of those peak values within 16 min (**Figures 2A–D**). Correspondingly, within 10 min of fentanyl administration, the model-predicted MV, V_T_, and RR decreased by up to 45, 22, and 13%, respectively, of their baseline values (**Figures 3B, D**; **Supplementary Figures S1A, B**, respectively) while ETCO_2_ increased by up to 17% (**Figure 3C**). In contrast, the predicted MAP remained relatively unchanged, with a ~2% decrease from its baseline value (**Figure 3A**, solid line). When fentanyl was administered with ketamine, within 1 min the predicted MAP increased by 34% of its baseline value, while MV predictions remained similar to those with fentanyl alone (**Figures 3A, B**, dashed lines). All the vital signs returned to within 10% of their baseline values ~30 min after fentanyl administration.

Based on our validation criterion, we established specific RMSE thresholds for each predicted variable (**Table 1**). All vital-sign predictions yielded RMSE values below their corresponding thresholds. For plasma fentanyl concentrations, the RMSE was below the threshold for *Study 5*; however, in the remaining three studies, the RMSE exceeded the threshold by 0.19–0.93 μg/L. This divergence likely occurred as concentrations approached zero, where the extremely small SEMs increased the relative impact of minor differences on the RMSE. On average, across the studies, we obtained RMSEs of 0.79 μg/L for plasma fentanyl concentration, 0.83 L/min for MV, 1.17 breaths/min for RR, 1.61 mmHg for MAP, 1.20 mmHg for ETCO_2_, and 0.09 L for V_T_. Notably, 95% of the vital-sign predictions and 45% of the plasma fentanyl predictions fell within two SEM of the experimental data. When we excluded *Study 6*, where for each fentanyl dose plasma concentration was only measured twice over the course of 60 min, ~60% of the predicted values fell within two SEM. Overall, while the quantitative performance for plasma fentanyl varied across studies, the model consistently and accurately captured the subsequent effects of fentanyl on vital signs in healthy individuals.

To validate the extended CR model for analgesia under hemorrhagic conditions, we compared its predictions of plasma fentanyl concentration following an administration of 50.00 μg/kg fentanyl in the absence of and after a 42% BV hemorrhage of swine in *Study 8* (**Figure 2E**, open and solid circles, respectively). The model predictions captured the overall trend, demonstrating that plasma fentanyl concentrations after hemorrhagic injury were, on average, ~64% higher than in the control condition (**Figure 2E**, dashed vs. solid line, respectively). Although the resulting RMSEs of 38.46 μg/L (hemorrhage) and 13.20 μg/L (control) for plasma fentanyl concentration exceeded their respective thresholds (28.72 μg/L and 10.61 μg/L) and, on average, 47% of the predictions fell within two SEM of the experimental data, the model qualitatively captured the fentanyl kinetics in healthy swine and the shift introduced by hemorrhage.

### 3.3 Physiological effects of analgesia under hypovolemia and airway obstruction

**Figure 4:**
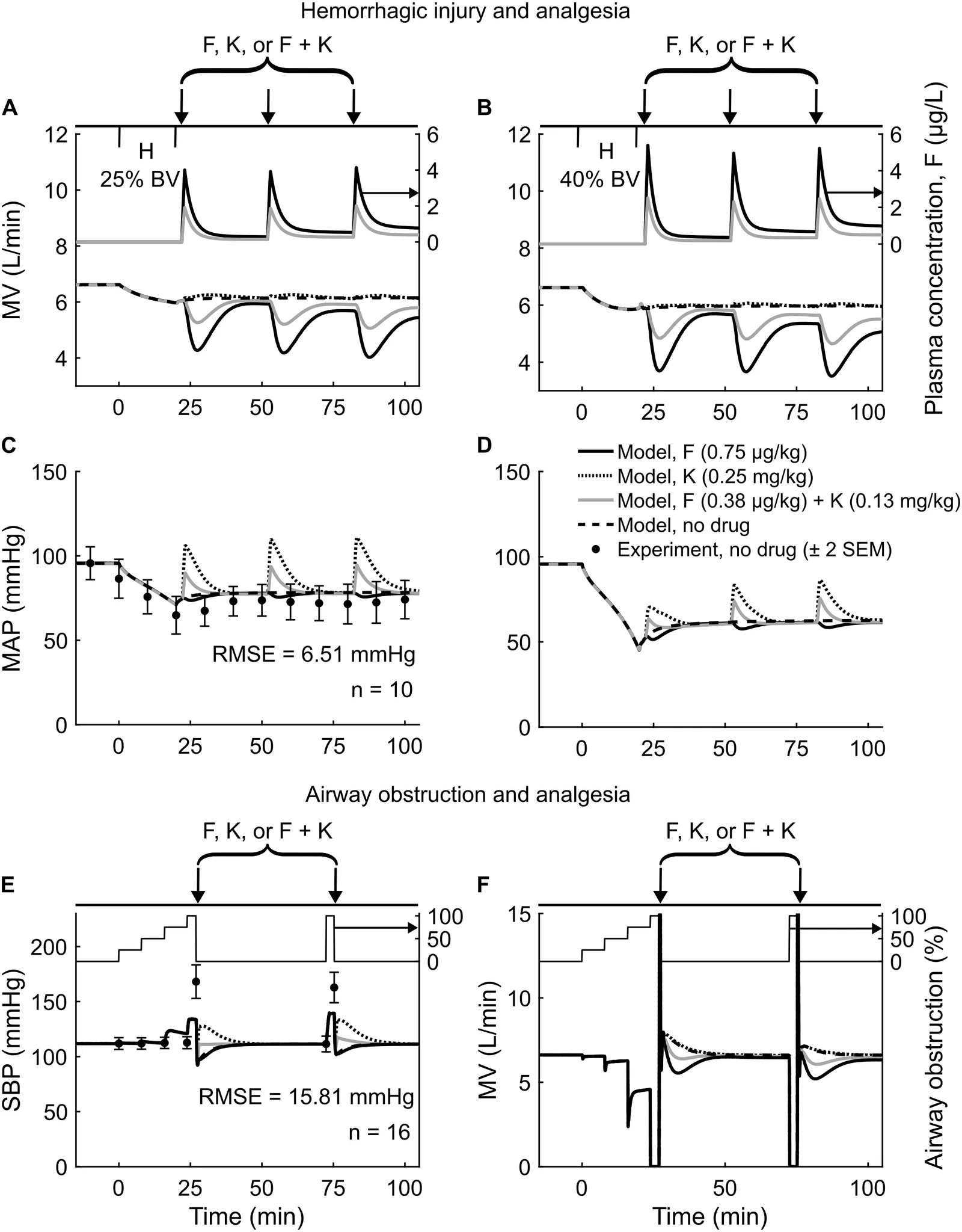
Results of simulations performed to investigate the effects of fentanyl (F) and ketamine (K) administered individually or in combination on **(A, B)** minute ventilation (MV) and **(C, D)** mean arterial pressure (MAP) following a hemorrhagic injury and on **(E)** systolic blood pressure (SBP) and **(F)** MV following an airway obstruction. In panels **(A, B)** the corresponding plasma fentanyl concentration changes are shown on the right y-axes. Panels **(A, C)** show the results for a 25% blood volume (BV) loss, and panels **(B, D)** show the results for a 40% BV loss. H represents the hemorrhage period. In panels **(E, F)**, the fractions of airway obstruction are shown on the right y-axes. The arrows at the top of each panel illustrate the time of each bolus drug administration for the experimental protocol. Error bars denote two standard errors of the mean (SEM). RMSE, root mean square error.

We investigated the effects of fentanyl and ketamine administration, individually and combined, on MAP, SBP, and MV during hemorrhage and airway obstruction using two sets of simulated scenarios, each performed with and without analgesia. First, we simulated a moderate hemorrhage (25% BV; **Figures 4A, C**) based on the study of Blackburn et al. (2021) and a severe hemorrhage (40% BV; **Figures 4B, D**). Starting at 2 min after the end of hemorrhage, and two more times at 30-min intervals, we simulated the administration of either fentanyl (doses ranging from 0.38 to 0.75 μg/kg) or ketamine (0.13 to 0.25 mg/kg), or both. In the absence of analgesia, consistent with the experimental study involving moderate hemorrhage (**Figure 4C**), our predictions of MAP decreased by 26% of its pre-hemorrhage baseline value and yielded an RMSE of 6.51 mmHg. In addition, MV (not reported in the experimental study) decreased by 10% of its baseline value, according to the model predictions (**Figure 4A**, dashed line). Following severe hemorrhage, for which we did not have experimental data, we observed similar trends for MV and MAP (**Figures 4B, D**, respectively, dashed lines).

Compared to no analgesia (**Figure 4**, dashed lines), for both moderate and severe hemorrhage, when fentanyl was administered alone, after each dose the predicted MAP and MV rapidly dropped, with peak decreases ranging from 3–5% and 28–37%, respectively, of their baseline values (**Figures 4A–D**, solid lines). When ketamine was administered alone, after each dose the predicted MAP and MV increased with peak increases ranging from 17–34% and 1–2%, respectively, of their baseline values (**Figures 4A–D**, dotted lines). When fentanyl and ketamine were administered together at half of their recommended doses (i.e., 0.38 μg/kg and 0.13 mg/kg, respectively), after each dose the predicted MAP increased with peak increases ranging from 10–17% and the MV dropped with peak decreases ranging from 13–20% of their respective baseline values (**Figures 4A–D**, solid gray lines). Interestingly, while the magnitude of the ketamine-induced increase in MAP diminished with hemorrhage severity, the fentanyl-induced decrease in MV became slightly more pronounced. For example, compared to no analgesia, when the same ketamine dose (0.25 mg/kg) was administered at 22 min, MAP increased by up to 32% of its baseline value for moderate hemorrhage but by only 17% for severe hemorrhage (**Figures 4C, D**, dotted lines). In contrast, for a fentanyl dose of 0.75 μg/kg administered at the same time point, MV decreased by up to 28% of its baseline value for moderate hemorrhage and by 34% for severe hemorrhage (**Figures 4A, B**, solid lines).

In the second set of scenarios, we simulated the airway obstruction protocol described in the study of Blackburn et al. (2019). Starting immediately at the end of a 100% airway obstruction (repeated twice in the experiment with a 50-min interval in between), we provided the same analgesics at the same doses and combinations that we simulated for the hemorrhagic scenarios. Without analgesia, consistent with experimental data (**Figure 4E**), after each instance of 100% obstruction, the predictions of SBP increased by ~22% of its baseline value, yielding an RMSE of 15.81 mmHg. In addition, MV decreased to zero during the 100% airway obstruction and returned to within 1% of its baseline value ~10 min after the removal of the obstruction (**Figure 4F**, dashed line).

Compared to no analgesia, when fentanyl was administered alone, after each dose the predicted SBP and MV decreased by up to 2% and 26%, respectively, of their baseline values (**Figures 4E, F**, solid lines). When fentanyl and ketamine were administered together, the predicted SBP increased while the predicted MV decreased by up to 13% of their respective baseline values (**Figures 4E, F**, solid gray lines). Finally, with only ketamine administration, both the predicted SBP and MV increased by up to 27% and 1%, respectively, of their baseline values (**Figures 4E, F**, dotted lines). Across the three analgesia protocols, SBP returned to within 1% of baseline ~1–9 min after drug administration, and MV returned within ~4–15 min.

## 4 Discussion

Given that fentanyl is one of two analgesics available on the battlefield for moderate to severe pain, we extended the CR model to incorporate its effects on vital signs, including MV, ETCO_2_, V_T_, RR, and MAP. This extension enables *in silico* investigations and hypotheses generation regarding the effects of fentanyl analgesia during complex battlefield trauma scenarios, especially hemorrhage and airway obstruction, and facilitates the generation of diverse synthetic datasets to train, test, and validate AI data-driven decision-support tools (Jin et al., 2024; Subramaniyan et al., 2025).

We assessed the performance of the extended CR model by comparing its predictions against six experimental studies in healthy humans and one study based on swine hemorrhagic-injury models, involving one to five intravenous fentanyl boluses (0.71–50.00 μg/kg) administered either alone or with ketamine (0.25 mg/kg). On average, we obtained reasonable prediction errors for the vital signs, including RMSEs of 0.83 L/min for MV, 1.20 mmHg for ETCO_2_, 1.17 breaths/min for RR, 0.09 L for V_T_, and 1.61 mmHg for MAP, which were within 3–12% of their baseline values (van Lemmen et al., 2025). Importantly, some of these RMSEs matched the reported accuracy of a representative Food and Drug Administration-cleared vital-sign monitor (i.e., the ZOLL Propaq M), with standard deviations of 2 mmHg for ETCO_2_, 1 breath/min for RR, and 3 mmHg for MAP, respectively (ZOLL Corporation, 2016). For plasma fentanyl concentration, the RMSEs following bolus administrations of 0.71–50.00 μg/kg were, on average, 0.79 μg/L in humans and 25.83 μg/L in swine, with 46% of the predicted values falling within two SEM of the experimental data. Overall, the model reasonably captured changes in plasma fentanyl concentration changes in healthy humans, healthy swine, and swine with hemorrhagic injury as well as the subsequent vital-sign changes in healthy humans.

Pain management in the prehospital environment is a key component of battlefield care, with over 70% of combat casualties requiring analgesia, often ketamine or fentanyl (Schauer et al., 2017; Hudson et al., 2023). Because we could not find any clinical or large-animal study data on the effect of fentanyl on vital signs after hemorrhagic injury or airway obstruction, we used the CR model to simulate such effects. In healthy individuals, the model adequately captured the primary effect of fentanyl administration, which is ventilatory depression exhibited by decreases in MV and RR (**Figures 3B, D, F**) and increases in ETCO_2_ (**Figure 3C**). Following moderate to severe hemorrhage, for the same fentanyl dose (0.75 μg/kg), the predicted peak plasma fentanyl concentrations were up to 55% higher than for simulated healthy cases due to the CO-induced shift in fentanyl kinetics (**Figures 4A, B**, right y-axes). In turn, this increase in fentanyl concentration in the plasma intensified the ventilatory depression effects, which increased with hemorrhage severity. For example, compared to no analgesia, administration of a fentanyl bolus of 0.75 μg/kg resulted in a decrease in MV by 19% without hemorrhage, 28% with 25% BV hemorrhage, and 34% with 40% BV hemorrhage (**Figures 4A, B**; 0% BV hemorrhage not shown). Interestingly, in similar simulations, we previously observed the opposite trend for ketamine analgesia with increases in hemorrhage severity (Kurian et al., 2025b). In those simulations, the ketamine-induced increases in MAP diminished as hemorrhage severity increased. Because ketamine relies on a healthy, active sympathetic nervous system to exert its cardiovascular effects, its impact on MAP is reduced when the system is already maximally activated to compensate for low blood (Kurian et al., 2025b). In contrast, fentanyl action does not strictly depend on the activation of the sympathetic nervous system. In fact, by inducing vasodilation and further lowering blood pressure, it can impair the effects of compensatory mechanisms for hemorrhage, which includes vasoconstriction (Watso et al., 2023).

For trauma-induced airway obstruction, our predictions showed that in the recovery period (~10 min) immediately following the removal of a 100% airway obstruction (where MV dropped to zero), compared to no analgesia, a 0.75-μg/kg fentanyl administration decreased MV by ~26% of its baseline value (**Figure 4F**, dashed vs. solid line). This indicates a slower recovery in comparison to a healthy individual with no airway obstruction, for whom the corresponding MV drop was ~19%. Given that fentanyl impairs MV in healthy individuals, administering it to trauma casualties with respiratory concerns necessitates careful oversight and frequent monitoring of respiratory vital signs.

Finally, we investigated the effects of co-administering fentanyl and ketamine *in silico* for both hemorrhage and airway obstruction. While both drugs are effective analgesics, and when administered together could synergistically increase analgesia (Tucker et al., 2005), their separate use is known to induce distinct adverse physiological effects. For example, both ketamine (at doses of ~0.3 mg/kg) and fentanyl (~1.1 μg/kg) are linked to adverse effects, such as hallucinations and ventilatory depression, respectively (Lee and Lee, 2016; Olofsen et al., 2022), although the probability of these effects decreases with lower doses of either drug (Gorlin et al., 2016; Teva Pharmaceuticals, 2023). Consistent with these observations, our simulations showed that reducing the fentanyl dose decreased the magnitude of adverse effects, such as ETCO_2_ increase, in healthy individuals (**Figure 3E**). Thus, we tested if combining lower doses of both drugs together could also mitigate their individual adverse vital-sign effects in trauma. Following the removal of 100% airway obstruction, the combined fentanyl and ketamine administration at half their recommended doses (i.e., 0.38 μg/kg and 0.13 mg/kg, respectively) returned SBP to baseline ~8–14 min faster than the full dose of ketamine alone and ~5–15 min faster than the full dose of fentanyl alone (**Figure 4E**, solid gray line). In addition, the combined administration also reduced the peak decrease in MV by 51% compared to fentanyl alone (**Figure 4F**, solid gray line).

Following moderate to severe hemorrhage (25–40% BV loss), the combination of fentanyl and ketamine reduced the adverse peak MV drop by 46–54% compared to fentanyl alone (**Figures 4A, B**, gray lines), although it also reduced ketamine’s beneficial increase in the MAP by 42–53% (**Figures 4C, D**, gray lines). In the future, we could use the model to further explore injury scenarios, fluid resuscitation, and varied analgesic regimens to predict the resulting vital-sign trajectories. The Pulse Physiology Engine is the only other model that can simulate the effects of both fentanyl and ketamine on vital signs, however, this model does not account for fentanyl PK changes induced by hemorrhage (Clipp et al., 2016). In addition, its predictions of the effects of ketamine on vital signs have only been qualitatively assessed, and its predictions of the effects of fentanyl in humans have only been quantitatively validated for a dose of 3.20 μg/kg, which is much higher than the TCCC recommended analgesic range of 0.50–1.00 μg/kg and is generally considered to be an overdose outside of a medical setting (Baird et al., 2024).

The extended CR model does have several limitations due to simplifying assumptions we had to make during model development. First, the model does not account for the underlying mechanisms by which pain itself affects the vital signs and how fentanyl might alter these effects because such mechanisms are currently unknown (Tousignant-Laflamme et al., 2005). Second, the fentanyl PD model equations were derived from healthy individuals, and we assumed these relationships also hold for trauma casualties. Third, because the original PD model was developed under iso-hypercapnic and iso-normic conditions, we recalibrated its parameters using data from realistic, non-controlled breathing conditions to improve physiological relevance. Despite this, the use of a simplified PD model may have resulted in an over- or underestimation of vital-sign changes, especially in simulations of hemorrhagic or airway-obstruction injuries. As experimental data or mechanistic models describing the effect of pain on vital signs or the effect of traumatic injuries on fentanyl PD become available, we can incorporate them to improve model accuracy. Fourth, when determining the incremental changes (ΔPaO_2_ and ΔPaCO_2_) required to match the target MV_PD_ in the algebraic transformation, we assumed that fentanyl induced proportional relative changes in PaO_2_ and PaCO_2_ compared to their baselines; this assumption may have affected the accuracy of the predicted fentanyl-induced MV changes. Finally, the current model considers only intravenous fentanyl administration. In scenarios where intravenous access is unavailable, fentanyl can be administered via intranasal or intramuscular routes, typically resulting in lower bioavailability of fentanyl (Foster et al., 2008). If necessary, in the future, we can further extend the CR model to include these fentanyl administration routes, using the corresponding PK models.

In summary, we extended and validated our previously developed CR model to account for vital-sign changes due to the administration of fentanyl analgesia. The extended model can now simulate the effects of both fentanyl and ketamine analgesia under different injury scenarios, allowing us to compare their effects on vital signs and generate new experimentally testable hypotheses for their use when treating trauma casualties. Finally, we can use the model to generate a diverse, physiologically relevant synthetic trauma dataset to enable the development of AI algorithms to help combat medics care for trauma casualties near the point of injury.

## Data Availability

The original contributions presented in the study are included in the article/**Supplementary Material**. Further inquiries can be directed to the corresponding authors.

## Glossary

[C_c_]: Fentanyl concentration in the central compartment (g•L^-1^)
[C_e_]: Fentanyl concentration at the effect site (g•L^-1^)
[C_p_]: Fentanyl concentration in the peripheral compartment (g•L^-1^)
c_0_: Intercept term in the quadratic function describing the relationship between relative changes in partial pressures of O_2_ and CO_2_ at steady state (Pgas_ss_) and target minute ventilation predicted by the fentanyl pharmacodynamic model (dimensionless)
c_1_: Coefficient of the linear term in the Pgas_ss_ quadratic function (dimensionless)
c_2_: Coefficient of the quadratic term in the Pgas_ss_ quadratic function (dimensionless)
C_50_: Fentanyl concentration for half-maximal respiratory depression (g•L^-1^)
Cl: Fentanyl elimination clearance (L•min^-1^)
Cl_0_: Fentanyl elimination clearance in healthy individuals (L•min^-1^)
CO: Cardiac output (L•min^-1^)
CO_B_: Baseline value of cardiac output (L•min^-1^)
ETCO_2_: End-tidal CO_2_ (mmHg)
HR: Heart rate (beats•min^-1^)
k_eo_: Fentanyl effect-site equilibration rate constant (min^-1^)
k_s,gas_: Smoothing rate constant for change in arterial partial pressures of CO_2_ and O_2_ (min^-1^)
MAP: Mean arterial pressure (mmHg)
MV_B_: Baseline value of minute ventilation (L•min^-1^)
MV_PD_: Target minute ventilation predicted by the fentanyl pharmacodynamic model (L•min^-1^)
PaCO_2,B_: Baseline value of arterial partial pressure of CO_2_ (mmHg)
PaO_2,B_: Baseline value of arterial partial pressure of O_2_ (mmHg)
Pgas_ss_: Relative changes in partial pressures of O_2_ and CO_2_ at steady state (dimensionless)
ΔPaCO_2_: Change in arterial partial pressure of CO_2_ provided as an input to controller (mmHg)
ΔPaO_2_: Change in arterial partial pressure of O_2_ provided as an input to controller (mmHg)
Q: Fentanyl intercompartmental clearance (L•min^-1^)
Q_0_: Fentanyl intercompartmental clearance in healthy individuals (L•min^-1^)
R_in_: Fentanyl administration rate (g•min^-1^)
RR: Respiratory rate (breaths•min^-1^)
SBP: Systolic blood pressure (mmHg)
V_c_: Fentanyl central compartment volume (L)
V_c0_: Fentanyl central compartment volume in healthy individuals (L)
V_P_: Fentanyl peripheral compartment volume (L)
V_p0_: Fentanyl peripheral compartment volume in healthy individuals (L)
V_T_: Tidal volume (L)
θ_Cl_: Gain quantifying CO effect on fentanyl elimination clearance (dimensionless)
θ_Q_: Gain quantifying CO effect on fentanyl intercompartmental clearance (dimensionless)
θ_Vc_: Gain quantifying CO effect on fentanyl central compartment volume (dimensionless)
θ_Vp_: Gain quantifying CO effect on fentanyl peripheral compartment volume (dimensionless)
