# Supplementary material for: A model to simulate cardio-respiratory responses to fentanyl analgesia after traumatic injury
Source: Front Physiol. 2026 Sep 4;17:1907767. doi: 10.3389/fphys.2026.1907767 (PMC13585624; doi:10.3389/fphys.2026.1907767)
Supplement: Supplementary file 1 [file DataSheet1.docx]

**A model to simulate cardio-respiratory responses to fentanyl analgesia after traumatic injury**

**Varghese Kurian^1,2^, Xin Jin^1,2^, Anders Wallqvist^1^, Jaques Reifman^1*^, and Sridevi Nagaraja^1,2*^**

^1^Department of Defense Biotechnology High Performance Computing Software Applications Institute, Defense Health Agency Research & Development, Medical Research and Development Command, Fort Detrick, MD, USA

^2^The Henry M. Jackson Foundation for the Advancement of Military Medicine, Inc., Bethesda, MD, USA

*** Correspondence:**
Sridevi Nagaraja, Ph.D. E-mail: snagaraja@bhsai.org

Jaques Reifman, Ph.D. E-mail: jaques.reifman.civ@health.mil

Keywords: Cardio-respiratory model, combat care, fentanyl analgesia, hemorrhage, pharmacokinetic-pharmacodynamic, pain management

**Supplementary Table S1.** List of newly added parameters in the extended CR model, with their assigned parameter number (P#), name, description, value, and units.

| **P#** | **Parameter** | **Description** | **Value***^a^* | **Unit** |
| --- | --- | --- | --- | --- |
| 1 | Cl_0_ | Elimination clearance of fentanyl in healthy individuals | 0.98 | L/min |
| 2 | V_c0_ | Central compartment volume of fentanyl in healthy individuals | 19.50 | L |
| 3 | Q_0_ | Intercompartmental clearance of fentanyl in healthy individuals | 3.51 | L/min |
| 4 | V_p0_ | Peripheral compartment volume of fentanyl in healthy individuals | 150.00 | L |
| 5 | k_eo_ | Effect site equilibration rate constant for fentanyl | 0.31 | min^-1^ |
| 6 | C_50_ | Fentanyl concentration for half-maximal respiratory depression | 5.20×10^-6^ | g/L |
| 7 | c_0_ | Intercept term in the Pgas_ss_ quadratic function | 2.83 | - |
| 8 | c_1_ | Coefficient of the linear term in the Pgas_ss_ quadratic function | -5.11 | - |
| 9 | c_2_ | Coefficient of the quadratic term in the Pgas_ss_ quadratic function | 2.28 | - |
| 10 | k_s,gas_ | Rate constant of the smoothing function of $\text{Δ}\text{Pa}\text{O}_{\text{2}}$ and $\text{Δ}\text{PaC}\text{O}_{\text{2}}$ | 0.30 | min^-1^ |
| 11 | θ_Cl_ | Gain quantifying the effect of CO on elimination clearance of fentanyl | 1.09 | - |
| 12 | θ_Vc_ | Gain quantifying the effect of CO on central compartment volume of fentanyl | 1.01 | - |
| 13 | θ_Q_ | Gain quantifying the effect of CO on intercompartmental clearance of fentanyl | 0.27 | - |
| 14 | θ_Vp_ | Gain quantifying the effect of CO on peripheral compartment volume of fentanyl | 0.75 | - |

CO: cardiac output; MAP: mean arterial pressure; MV: minute ventilation; Pgas_ss_: an intermediate variable in the algebraic transformation; ΔPaO_2_: change in partial pressure of arterial oxygen given as input to the neuronal controller; ΔPaCO_2_: change in partial pressure of arterial carbon dioxide given as input to the neuronal controller.

*^a^*We obtained Parameters 1−4 from Yassen et al. (2007). We calibrated Parameters 5, 6, and 10 using the data of Mildh et al. (1998) and Mildh et al. (2001). We estimated Parameters 7−9 as coefficients of a quadratic function and estimated Parameters 11−14 using the pharmacokinetic models of Han et al. (2007).

**Supplementary Figure S1.** Comparison of the extended cardio-respiratory (CR) model’s vital-sign predictions with the corresponding experimental data from *Study 5*. (**A**) Tidal volume. (**B**) Respiratory rate (RR). We show only the first 180 min for visualization purposes, however, we used the entire 242-min dataset for calculating the root mean square error (RMSE) and threshold. The arrows at the top of each panel illustrate the time and dose of each bolus fentanyl administration for the experimental protocol.
